# Effects of Intensive Blood Pressure Lowering on Cardiovascular and Renal Outcomes: A Systematic Review and Meta-Analysis

**DOI:** 10.1371/journal.pmed.1001293

**Published:** 2012-08-21

**Authors:** Jicheng Lv, Bruce Neal, Parya Ehteshami, Toshiharu Ninomiya, Mark Woodward, Anthony Rodgers, Haiyan Wang, Stephen MacMahon, Fiona Turnbull, Graham Hillis, John Chalmers, Vlado Perkovic

**Affiliations:** 1 The George Institute for Global Health, The University of Sydney, Sydney, Australia; 2 Renal Division, Department of Medicine, Peking University First Hospital, Beijing, China; 3 Department of Epidemiology, Johns Hopkins University, Baltimore, Maryland, United States of America; 4 Department of Medicine and Clinical Science Graduate School of Medical Sciences, Kyushu University, Japan; Barts and The London School of Medicine and Dentistry, United Kingdom

## Abstract

**Background:**

Guidelines recommend intensive blood pressure (BP) lowering in patients at high risk. While placebo-controlled trials have demonstrated 22% reductions in coronary heart disease (CHD) and stroke associated with a 10-mmHg difference in systolic BP, it is unclear if more intensive BP lowering strategies are associated with greater reductions in risk of CHD and stroke. We did a systematic review to assess the effects of intensive BP lowering on vascular, eye, and renal outcomes.

**Methods and Findings:**

We systematically searched Medline, Embase, and the Cochrane Library for trials published between 1950 and July 2011. We included trials that randomly assigned individuals to different target BP levels.

We identified 15 trials including a total of 37,348 participants. On average there was a 7.5/4.5-mmHg BP difference. Intensive BP lowering achieved relative risk (RR) reductions of 11% for major cardiovascular events (95% CI 1%–21%), 13% for myocardial infarction (0%–25%), 24% for stroke (8%–37%), and 11% for end stage kidney disease (3%–18%). Intensive BP lowering regimens also produced a 10% reduction in the risk of albuminuria (4%–16%), and a trend towards benefit for retinopathy (19%, 0%–34%, *p* = 0.051) in patients with diabetes. There was no clear effect on cardiovascular or noncardiovascular death. Intensive BP lowering was well tolerated; with serious adverse events uncommon and not significantly increased, except for hypotension (RR 4.16, 95% CI 2.25 to 7.70), which occurred infrequently (0.4% per 100 person-years).

**Conclusions:**

Intensive BP lowering regimens provided greater vascular protection than standard regimens that was proportional to the achieved difference in systolic BP, but did not have any clear impact on the risk of death or serious adverse events. Further trials are required to more clearly define the risks and benefits of BP targets below those currently recommended, given the benefits suggested by the currently available data.

*Please see later in the article for the Editors' Summary.*

## Abstract 2

In a systematic review and meta-analysis Vlado Perkovic and colleagues investigate whether more intensive blood pressure lowering regimens are associated with greater reductions in the risk of major cardiovascular events and end stage kidney disease.

## Editors' Summary

**Background:**

About a third of US and UK adults have high blood pressure (hypertension). Although hypertension has no obvious symptoms, it can lead to heart attacks, stroke, and other forms of cardiovascular disease, to kidney failure, and to retinopathy (blindness caused by damage to the blood vessels in the back of the eye). Hypertension is diagnosed by measuring blood pressure (BP)—the force that blood moving around the body exerts on the inside of large blood vessels. BP is highest when the heart is pumping out blood (systolic BP) and lowest when it is refilling with blood (diastolic BP). A normal adult BP is defined as a systolic BP of less than 130 millimeters of mercury (mmHg) and a diastolic BP of less than 85 mmHg (a BP of 130/85). A reading of more than 140/90 indicates hypertension. Many factors affect BP, but overweight people and individuals who eat fatty or salty food are at high risk of developing hypertension. Mild hypertension can be corrected by making lifestyle changes, but people often take antihypertensive drugs to reduce their BP.

**Why Was This Study Done?:**

Doctors usually try to reduce the BP of their hypertensive patients to 140/90 mmHg. However, some treatment guidelines now advocate a target BP of 130/80 mmHg for individuals at high risk of life-threatening cardiovascular events, such as people with diabetes or kidney impairment. But does more intensive BP lowering actually reduce the risk of heart attacks and stroke? Although placebo-controlled randomized trials of BP lowering have suggested that a 10 mmHg fall in systolic BP is associated with a 22% reduction in the risk in coronary heart disease and a 41% reduction in the risk of stroke, it is unclear whether intensive BP lowering strategies are associated with greater reductions in the risk of cardiovascular disease than standard strategies. In this systematic review (a search that uses predefined criteria to identify all the research on a given topic) and meta-analysis (a statistical method for combining the results of studies), the researchers investigate the effects of intensive BP lowering on cardiovascular, eye, and renal outcomes.

**What Did the Researchers Do and Find?:**

The researchers identified 15 randomized controlled trials in which more than 37,000 participants were randomly assigned to antihypertensive drug-based strategies designed to achieve different target BPs. On average, the more intensive strategies reduced the BP of participants by 7.5/4.5 mmHg more than the less intensive strategies. Compared to standard BP lowering strategies, more intensive BP lowering strategies reduced the risk of major cardiovascular events (a composite endpoint comprising heart attack, stroke, heart failure, and cardiovascular death) by 11%, the risk of heart attack by 13%, the risk of stroke by 24%, the risk of end-stage kidney disease by 11%, and the risk of albuminuria (protein in the urine, a sign of kidney damage) by 10%. There was also a trend towards a reduced risk for retinopathy with more intensive BP lowering but no clear reduction in cardiovascular or noncardiovascular deaths. Finally, aiming for a lower BP target did not increase the rate of drug discontinuation or the risk of serious adverse events apart from hypotension (very low BP).

**What Do These Findings Mean?:**

These findings suggest that, although intensive BP lowering regimens have no clear effect on the risk of death, they may provide greater protection against cardiovascular events than standard BP lowering regimens. Indeed, the researchers calculate that among every thousand hypertensive patients with a high cardiovascular risk, more intensive BP lowering could prevent two of the 20 cardiovascular events expected to happen every year. Although intensive BP lowering did not seem to increase the risk of severe adverse effects, the accuracy of this finding is limited by inconsistent reporting of adverse events in the trials included in this study. Moreover, because most of the trial participants had additional risk factors for cardiovascular events such as diabetes and chronic kidney disease, these findings may not be generalizable to people with hypertension alone. Thus, although this study suggests that a target BP of 130/80 is likely to produce an additional overall benefit compared to a target of 140/90, more trials are needed to confirm this conclusion and to determine the best way to reach the lower target.

**Additional Information:**

Please access these websites via the online version of this summary at http://dx.doi.org/10.1371/journal.pmed.1001293.

- The US National Heart Lung and Blood Institute has patient information about high blood pressure (in English and Spanish)
- The American Heart Association provides information on high blood pressure and on cardiovascular diseases (in several languages); it also provides personal stories from people dealing with high blood pressure
- The UK National Health Service (NHS) Choices website also provides detailed information for patients about hypertension, cardiovascular disease and kidney disease; the NHS Local website has a collection of personal stories about hypertension and a series of films that explain hypertension
- MedlinePlus provides links to further information about high blood pressure, heart disease, stroke, and kidney disease (in English and Spanish)

## Introduction

Cohort studies show continuous positive associations of blood pressure (BP) with cardiovascular risk with no evidence of a threshold at BP levels down to 110/70 mmHg [1]–[3]. Large-scale placebo-controlled randomised trials of BP lowering have achieved reductions in risk of 22% for coronary heart disease (CHD) and 41% for stroke for every 10 mmHg lower BP achieved, that the risk reduction correlates almost exactly with that anticipated from epidemiological studies [4],[5]. In addition, in trials of BP lowering versus control, the greater BP reductions achieved by combination treatment have produced greater risk reductions than those obtained for monotherapy [4]. Finally, in trials comparing different BP lowering agents, the trials with larger BP differences have also resulted in greater differences in effects on clinical outcomes [5].

As trial evidence has accumulated, the BP targets recommended by guideline groups have been progressively lowered and intensive BP lowering is now widely advocated for individuals at high cardiovascular risk [6]–[10]. These recommendations are, however, still debated in recent national guidelines [11]–[14], due in part to some observational analyses that have reported associations of low BP with increased coronary disease risk. However, it is not certain whether this is causal or represents the effects of preclinical disease both lowering BP and independently increasing risk. A 2003 systematic overview that included five trials and about 22,000 individuals concluded that more intensive BP lowering provided significantly greater cardiovascular protection but did not address a key question about the effects of targeting different BP levels [5]. More recently, a Cochrane review using different trial inclusion criteria reported no greater benefit for intensive regimens targeting BP levels of <135/85 mmHg compared to standard BP targets [15].

The completion, in the last few years, of three large new trials evaluating the effects of different intensities of BP lowering on cardiovascular outcomes provides an opportunity to re-assess the evidence for lower BP targets [16]–[18]. In this systematic review, we sought to synthesize all the available clinical trial data and better define the balance of risks and benefits associated with different intensities of BP lowering.

## Methods

### Data Sources and Searches

We performed a systematic review of the literature in line with the approach recommended by the PRISMA statement for the conduct of meta-analyses of intervention studies (Text S1) [19]. Relevant studies were identified by searching the following data sources: MEDLINE via Ovid (from 1950 through July 2011), EMBASE (from 1966 through July 2011), and the Cochrane Library database, using relevant text words and medical subject headings that included all spellings of antihypertensive agents, target BP, intensive BP treatment, intensive BP control, strict BP treatment, strict BP control, tight BP treatment, and tight BP control (see Text S2). The search was limited to randomized controlled trials with at least 6 mo follow-up, but without age or language restriction. Reference lists from identified trials and review articles were manually scanned to identify any other relevant studies. The ClinicalTrials.gov website was also searched for randomized trials that were registered as completed but not yet published.

### Study Selection

The literature search, data extraction, and quality assessment were conducted independently by two authors using a standardized approach (JL and PE). All completed randomized controlled trials that compared more versus less intensive BP targets with pharmacological BP lowering agents were eligible for inclusion, including those that included participants with hypertension, high vascular/renal risk, or both.

### Data Extraction and Quality Assessment

Published reports were obtained for each trial and standard information was extracted into a spreadsheet. The data sought included baseline patient characteristics (age, gender, mean systolic and diastolic BP levels, history of diabetes, history of hypertension, and chronic kidney disease [CKD]), BP control target in each arm, BP lowering agents, follow-up duration, mean reduction of systolic and diastolic BP during the trial, outcome events, and adverse events. Study quality was judged by the proper conduct of randomization, concealment of treatment allocation, similarity of treatment groups at baseline, the provision of a description of the eligibility criteria, completeness of follow-up, and use of intention-to-treat analysis. The Cochrane Collaboration's tool was used for assessing risk of bias. Any disagreement in abstracted data was adjudicated by a third reviewer (VP).

### Outcomes

The primary outcome was major cardiovascular events defined as a composite of myocardial infarction, stroke, heart failure, and cardiovascular death. Secondary outcomes were each individual component of the composite primary outcome, all-cause mortality, end stage kidney disease (ESKD), and adverse outcomes. Progression of albuminuria (defined as new onset of micro-/macro-albuminuria or microalbuminuria to macroalbuminuria) and retinopathy (retinopathy progression ≥2 steps) were also recorded for trials done in patients with diabetes.

### Data Synthesis and Analysis

Individual patient data (IPD) were not available for the studies in this analysis so tabular data were used. Individual study relative risk (RR) ratios and 95% CIs were calculated for each outcome before pooling. Where continuous scales of measurement were used to assess the effects of treatment (BP), then the mean difference (MD) was used. Summary estimates of RR ratios or MD were obtained using a random effects model. The percentage of variability across studies attributable to heterogeneity beyond chance was estimated using the *I* ^2^ statistic [20]. Potential publication bias was assessed using the Egger test and represented graphically using Begg funnel plots of the natural log of the RR versus its standard error [21]. Evidence for heterogeneity in estimates of treatment effect attributable to the baseline characteristics of the trials was explored by comparing summary results obtained from subsets of studies grouped by number of patients, cardiovascular event rate, age, diabetes, BP target, and BP level at baseline. A two-sided *p*-value less than 0.05 was considered statistically significant and statistical analyses were performed using STATA version 10.1 (Stata).

## Results

### Search Results and Characteristics of Included Studies

**Figure 1:**
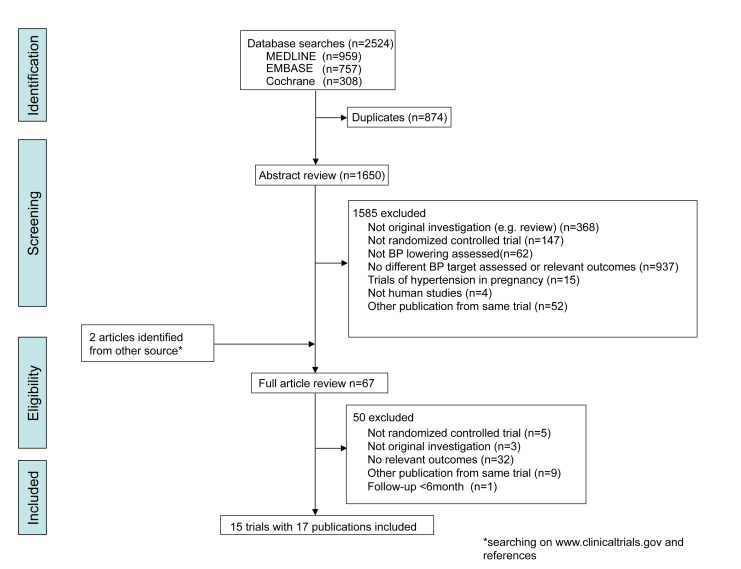
Identification process for eligible studies.

The literature search yielded 1,650 articles of which 67 were reviewed in full text and from which 15 randomized controlled trials reported in 17 publications were identified (Figure 1) [16]–[18],[22]–[35]. These trials provided information on a total of 37,348 patients among whom 1,984 major cardiovascular events were reported from ten studies, 1,584 deaths from 15 studies, and 941 ESKD events from eight studies. All the trials were open designs with few patients lost to follow-up (0%–4.9%). Mean study follow-up duration ranged from 1.6 to 12.2 y. The reported trial quality varied substantially (Table S1).

Among the 15 trials, five (*n* = 6,960) enrolled only patients with diabetes [16],[23],[25],[26],[31] and six specifically recruited participants with CKD (*n* = 2,734) (Table 1) [22],[29],[30],[33]–[35]. One of the studies was done in children with CKD and hypertension (*n* = 385, with mean age 11.5 y) [33].

**Table 1:** Characteristics of the studies included.

Study/Author	Inclusion Criteria	Baseline BP (mmHg)	BP Target in Active Group (mmHg)	BP Target in Control Croup (mmHg)	Design Country of Origin	Duration of Follow-up (y)	*n* Patients	Mean Age (y)	Female (%)	Diabetes Mellitus	Primary Endpoint	*n* Primary Endpoint	*n* Major CV Event	Definition of Major CV Event
Toto RD et al. 1995 [22]	Hypertensive nephrosclerosis with Scr>1.6 mg/dl or GFR<70 ml/min.1.73 m^2^	123.1/76.5	Diastolic BP 65–80	Diastolic BP 85–95	Randomised unspecified number of centres/US	3.4	77	55.7	37.7	Excluded	Rate of decline in GFR	NA (9 in ESKD)	NR	NR
HOT 1998 [24]	Hypertension with diastolic BP 100–115 mmHg	169.7/105.4	Diastolic BP<80	Diastolic BP<85 or <90	Randomised multicentre/Sweden, Italy, Canada, US, France, Germany	3.8	18,790	61.5	47	1,501 (8%)	Major CV event	683	683	CV death nonfatal MI/stroke
UKPDS-HDS 1998 [23]	Newly diagnosed type 2 diabetes with hypertension	159.3/94	BP<150/85	BP<180/105	Randomised multicentre/UK	8.4	1,148	56	44.5	1,148 (100%)	(a) clinical endpoint related to diabetes; (b) death related to diabetes; (c) death from all cause	(a) 429; (b) 144; (c) 217	271	CV death nonfatal MI/stroke
ABCD (H) 2000 [25]	Type 2 diabetes with diastolic BP≥90 mmHg	155/98	Diastolic BP<75	Diastolic BP 80–89	Randomised multicentre/US	5	470	57.9	32.6	470 (100%)	Change of creatinine clearance	NA	75	CV death nonfatal MI/stroke, admission for heart failure
ABCD (N) 2001 [26]	Type 2 diabetes with normotension (diastolic BP 80–89 mmHg)	136.4/84.4	Diastolic BP reduction 10 mmHg from baseline	Diastolic BP 80–89 mmHg	Randomised multicentre/US	5.3	480	59.1	45.5	480 (100%)	Change of creatinine clearance	NA	76	CV death nonfatal MI/stroke, admission for heart failure
Schrier R 2002 [34]	ADPKD patients with hypertension, left ventricular hypertrophy, and creatinine clearance >30 ml/min per 1.73 m^2^	142.5/95.5	<120/80	135–140/85–90	Randomized single centre/US	7	75	41.1	45	NR	Not specified	NA	NA	
AASK 2010 [35]	African American with hypertension and GFR 20–65 ml/min.1.73 m^2^ and no other identified causes of renal insufficiency	150.5/95.5	Mean BP<92 mmHg	Mean BP 102–107 mmHg	Randomised multicentre/US	8.8–12.2	1,094	54.6	38.8	Excluded	Doubling of serum creatinine, ESKD, or death	567	225	CV death nonfatal MI/stroke, admission for heart failure
MDRD 2005 [29]	CKD with Scr 1.4–7.0 mg/dl in male or 1.2–7.0 mg/dl in female	130.5/80	Mean BP<92 mmHg	Mean BP<107 mmHg	Randomised multicentre/US	10.6	840	51.7	40	43 (5.1%)	Rate of decline in GFR	NA (ESKD 554)	NR	NR
REIN-2 2005 [30]	Nondiabetic nephropathy with proteinuria 1–3 g/d and GFR<45 ml/min.1.73 m^2^ or proteinuria >3 g/d and GFR<70 ml/min.1.73 m^2^	136.7/84.1	BP<130/80	Diastolic BP<90	Randomised multicentre/Italy	1.6	338	53.9	25.7	Excluded	ESKD	72	9	CV death nonfatal MI/stroke,admission for heart failure
ABCD(2V) 2006 [31]	Type 2 diabetic patients with BP<140/80–90 mmHg without overt albuminuria	126/84	Diastolic BP<75 mmHg	Diastolic BP<90 mmHg	Randomized single center/US	1.9	129	56.1	32.6	129 (100%)	Change of creatinine clearance and UAE	NA	5	NR
JATOS 2008 [32]	Elderly hypertensive patients with 65–85 y and systolic BP>160 mmHg	171.6/89.1	Systolic BP<140	Systolic BP<160	Randomised multicentre/Japan	2	4,418	73.6	61.1	521 (11.8%)	Cardiovascular event and renal failure	172	100	CV death nonfatal stroke and nonfatal MI
Cardio-Sis 2009 [17]	Nondiabetic patients with systolic BP>150 mmHg and at least one additional risk factor	163/89.6	Systolic BP<130	Systolic BP<140	Randomised multicentre/Italy	2	1,111	67	59	1111 (100%)	Electrocardiographic left ventricular hypertrophy	137	49	Death, MI, hospitalization for heart failure, angina, or coronary revasculisation
ESCAPE 2009 [33]	CKD with age 3–18 y and GFR 15–80 ml/min.1.73 m^2^ whose 24-h mean BP elevated or controlled by antihypertensive agents	118.3/73.0	24-h mean BP below the 50th percentile	24-h mean BP in the 50th–95th percentile	Randomised multicentre/Germany, Italy, Poland, Turkey, France, Sweitzerland	5	385	11.5	41	NR	Decline of 50% in GFR or ESKD	115	NR	NR
ACCORD 2010 [16]	Type 2 diabetic patients with 40 y older and cardiovascular disease or 55 y older with risk factors for cardiovascular disease	139.2/76.0	Systolic BP<120 mmHg	Systolic BP<140 mmHg	Randomised multicentre/US, Canada	4.7	4,733	62.2	47.7	4,733 (100%)	Major CV event	439	439	CV death nonfatal stroke and nonfatal MI
VALISH 2010 [18]	Age≥70 and ≤85 y with isolated systolic hypertension (BP>160 systolic and <90 mmHg diastolic)	169.6/81.4	Systolic BP<140 mmHg	Systolic BP 140–150 mmHg	Randomised multicentre/Japan	2.85	3,260	76.1	62.5	NR	Composite of CV event and renal dysfunction	99	69	CV death, nonfatal stroke, and nonfatal myocardial infarction

ADPKD, autosomal dominant polycystic kidney disease; CV, cardiovascular; GFR, glomerular filtration rate; MI, myocardial infarction; NA, not available; NR, not reported.

Two trials (*n* = 609) recruited diabetic patients without hypertension with mean baseline BP 136/84 and 126/84 mmHg [26],[31]. The other 12 trials (*n* = 36,664) recruited patients with hypertension [16]–[18],[22]–[30],[32],[35]. The mean baseline BP levels in the trials of adults were between 131/80 and 172/105 mmHg and 109/64 in the trial done in children.

The BP targets varied substantially between trials. The three most conservative trials sought to meet or better intensive group targets of 140–150 mmHg systolic and 85–90 mmHg diastolic [18],[23],[32], while the most aggressive studies had systolic BP targets that were 20–30 mmHg below these levels [16],[17],[30],[34]. Four trials had diastolic BP targets below 80 mmHg [24]–[26],[31]. Across all trials, the weighted mean follow-up difference in BP between the more versus less intensively treated groups was 7.5 mmHg for systolic BP and 4.5 mmHg for diastolic BP.

### Effects of Intensive BP Lowering Regimens

#### Major cardiovascular events

**Figure 2:**
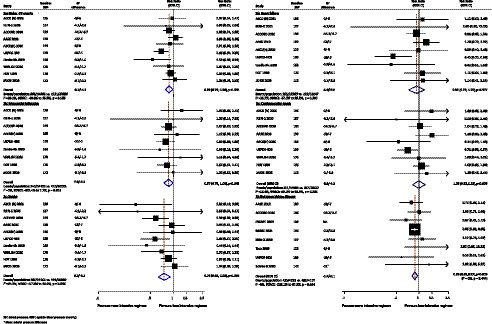
Effect of intensive BP lowering on risk of major cardiovascular events (a), myocardial infarction (b), and stroke (c). Boxes and horizontal lines represent RR and 95% CI for each trial. Size of boxes is proportional to weight of that trial result. Diamonds represent the 95% CI for pooled estimates of effect and are centered on pooled RR.

Data regarding the effects of intensive BP regimens on major cardiovascular events were available from ten trials including 35,842 participants and 1,984 cardiovascular events (Figure 2a). Overall, more intensive BP lowering regimens produced an 11% (RR 0.89, 95% CI 0.79–0.99, *p* = 0.036) reduction in the risk of major cardiovascular events compared to less intensive regimens with no evidence of heterogeneity in the magnitude of the effect across the included studies (*I* ^2^ = 28.2%, *p* = 0.185).

#### Cause-specific vascular outcomes

**Figure 3:**
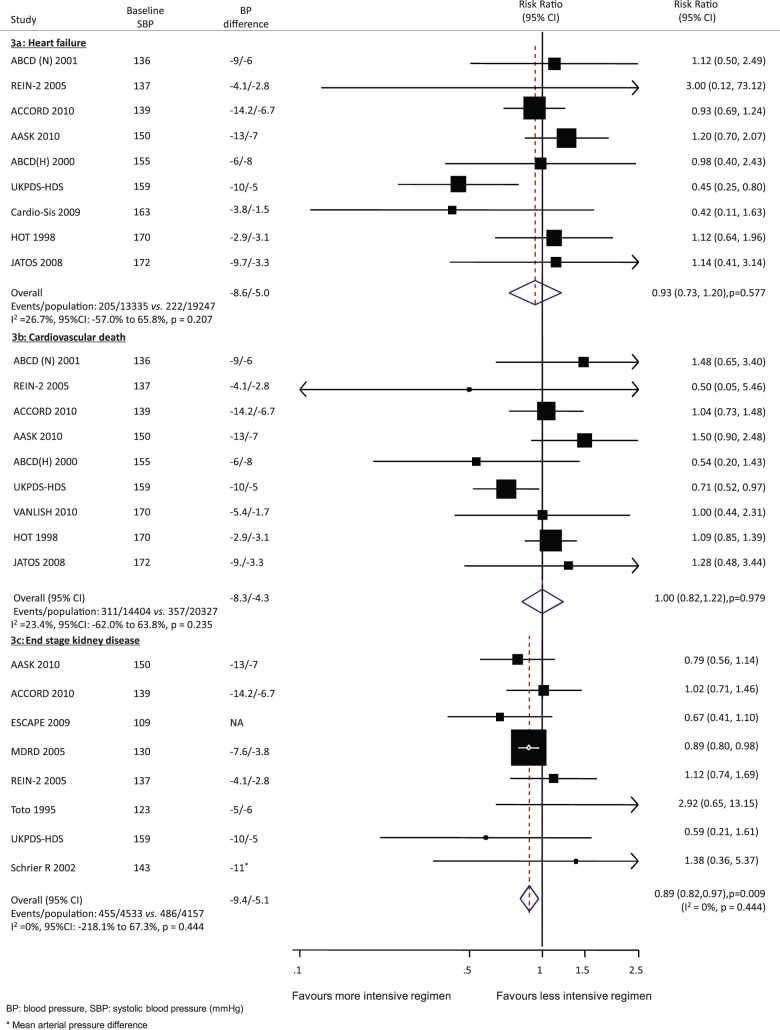
Effect of intensive BP lowering on risk of heart failure (a), cardiovascular death (b), and end stage kidney disease (c). Boxes and horizontal lines represent RR and 95% CI for each trial. Size of boxes is proportional to weight of that trial result. Diamonds represent the 95% CI for pooled estimates of effect and are centered on pooled RR.

Myocardial infarction was reported by nine trials including 34,748 participants among whom 756 events were observed (Figure 2b). More intensive BP lowering therapy reduced the risk of myocardial infarction by 13% (RR 0.87, 95% CI 0.75–1.00, *p* = 0.049). There were ten trials (35,842 participants) that reported 726 stroke events and nine trials (32,582 participants) reported 427 occurrences of heart failure. More intensive BP regimens were associated with a 24% (RR 0.76, 95% CI 0.63–0.92, *p* = 0.004) lower risk of stroke (Figure 2c), but there was no clearly apparent beneficial effect for heart failure (RR 0.93, 95% CI 0.73–1.20, *p* = 0.577) (Figure 3a). As illustrated in Table 2, the magnitudes of the risk reductions observed for stroke (24%, 95% CI 8%–37%) and CHD (13%, 95% 0%–25%) in this meta-analysis were directly comparable to those anticipated from large cohort studies (stroke 27% and CHD 19% with a 7.5-mmHg systolic BP difference) [2]. The effects were also consistent with the observed effects of a meta-analysis of trials comparing BP lowering agents against control that standardized to a 7.5-mmHg systolic BP difference between randomized groups (stroke 33% and CHD 17%) [4].

**Table 2:** Comparison of expected and observed effects of a 7.5-mmHg systolic blood pressure difference on coronary heart disease, stroke, and heart failure.

Relative Risk Reduction	CHD	Stroke
Expected^a^ from cohort studies	19%	27%
Observed^a^ in trials of BP lowering versus control	17%	33%
Observed in trials of more versus less BP lowering	13%	24%

a The associations observed in cohort studies [2] and the reductions shown in trials of BP lowering versus control [4] are shown, standardized to the 7.5-mmHg systolic difference seen in the current meta-analysis (e.g., previous trials showed a RR for stroke of 0.59 with a 10 mmHg systolic reduction, so one would expect a 33% reduction for 7.5 mmHg lower systolic, as 0.59^7.5/10^ = 0.67).

#### Fatal events

There was no clear effect of more intensive BP lowering on the risk of cardiovascular death (RR 1.00, 95% CI 0.82–1.22, *p* = 0.979) (Figure 3b), noncardiovascular death (RR 0.97, 95% CI 0.84–1.11, *p* = 0.621), or all-cause death (RR 1.00, 95% CI 0.91 to 1.10, *p* = 0.995) as compared with less intensive BP control, with CIs that were compatible with modest effects in either direction.

#### End stage kidney disease

Eight trials including 8,690 participants recorded 941 ESKD outcomes. Compared to less intensive BP lowering, a more intensive regimen reduced the risk of ESKD by 11% (RR 0.89, 95% CI 0.82–0.97, *p* = 0.009) without evidence of heterogeneity (*I* ^2^ = 0%, *p* = 0.444) (Figure 3c).

#### Microvascular events in diabetes

**Figure 4:**
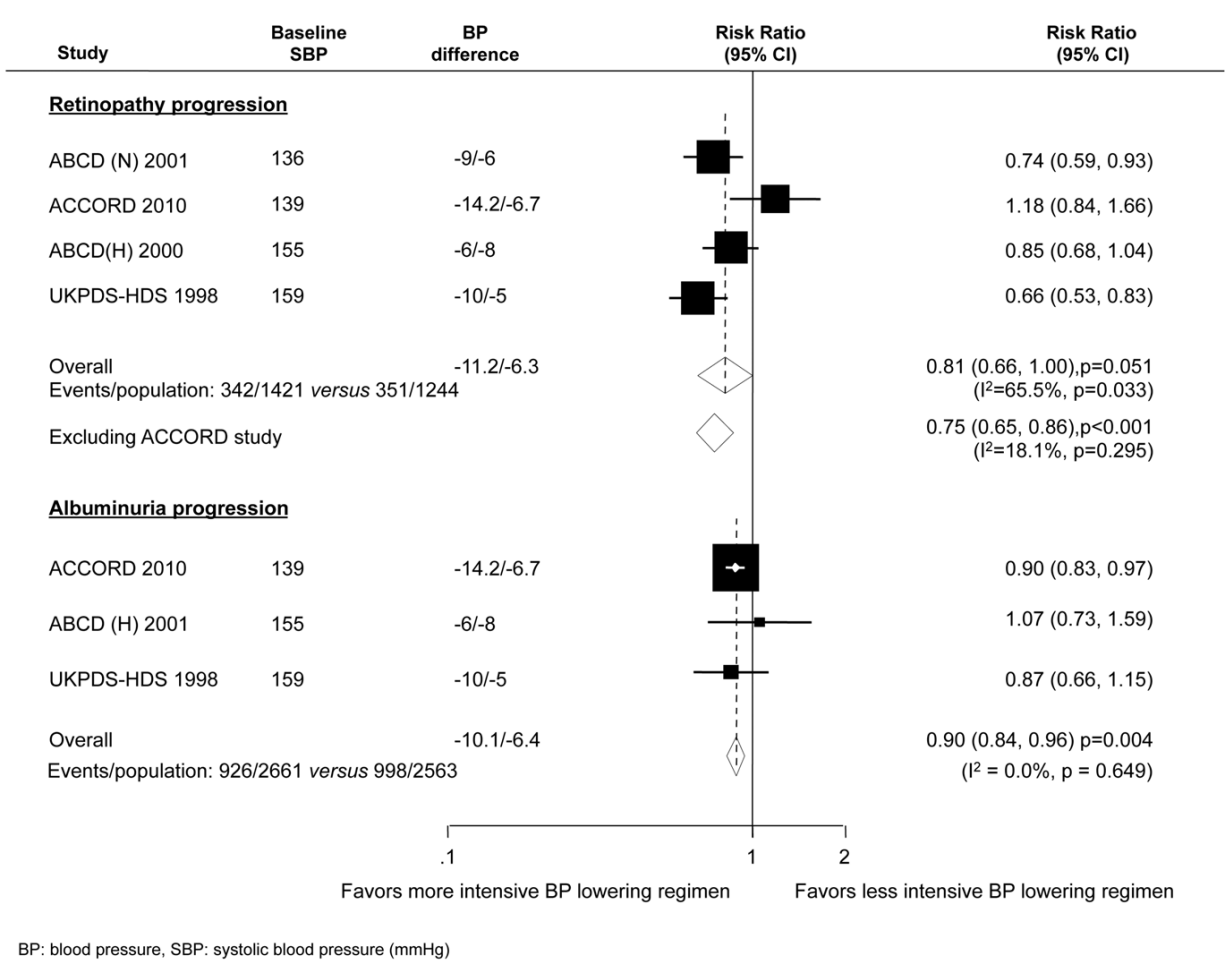
Effect of intensive BP lowering on the risk of microvascular outcomes in diabetes.

Three trials reported data on progression of albuminuria (5,224 participants and 1,924 events) and more intensive BP control reduced the risk of albuminuria progression by 10% (RR 0.90, 95% CI 0.84–0.96, *p* = 0.004) with no evidence of heterogeneity (*I* ^2^ = 0.0%, *p* = 0.649) (Figure 4). Progression of retinopathy was reported by four trials with 2,665 participants and 693 events. There was a borderline significant reduction in retinopathy with more intensive BP lowering (RR 0.81, 95% CI 0.66–1.00, *p* = 0.051) but substantial heterogeneity in the magnitude of the effect across the included studies (*I* ^2^ = 65.5%, *p* = 0.033) (Figure 4) mostly attributable to the ACCORD result. A sensitivity analysis excluding ACCORD resulted in a risk reduction of 25% (RR 0.75, 95% CI 0.65–0.86, *p*<0.001) with a much reduced *I* ^2^ value of 18.1%. Of note, there were significant imbalances in a number of the baseline characteristics between randomized arms in this substudy of ACCORD [4].

### Potential Harms of Treatment

Data on adverse outcomes potentially associated with treatment were collected from the trials but were inconsistently reported (Table 3). Five trials reported data on severe adverse events (SAEs) (9,827 participants and 564 events) [16]–[18],[30],[33] and four trials on total adverse events (AEs) (9,174 participants and 1,877 events) [17],[18],[33],[36] showing no clear effect of more intensive BP lowering compared to less intensive BP lowering on SAEs (RR 1.19, 0.88–1.61, *p* = 0.250) or AEs (RR 0.99, 0.92–1.08, *p* = 0.844). Four trials [16],[17],[29],[33] reported hypotension outcomes (5,118 participants, with 76 versus 16 events) with more intensive BP control greatly increasing the risk of hypotension (RR 4.16, 95% CI 2.25–7.70, *p*<0.001) and showing an adverse trend towards severe hypotension (RR 2.19, 95% CI 0.03–164.77, *p* = 0.723) although the annual rate of severe hypotension was very low (0%–0.15%) [16],[33]. More intensive BP control did not clearly increase the risk of dizziness (three trials, 6,629 participants, and 413 events; RR 1.15, 95% CI 0.95–1.38, *p* = 0.148) [16],[17],[33]. Finally, there was no clear difference detected in the rate of drug discontinuation between the more intensive and less intensive treated groups in the four trials that reported data (9,874 participants, 340 events; RR 0.96, 95% CI 0.79–1.16) [16],[17],[32],[33].

**Table 3:** Adverse events between more intensive and less intensive BP lowering regimen.

Adverse Event	Study	Participants	Events Rate* (More/Less Intensive)	RR (95% CI)	*p*-Value
Total Severe AEs [16]–[18],[30],[33]	5	9,827	309 (1.7)/255(1.4)	1.19 (0.88–1.61)	0.250
Total AEs [17],[18],[33],[36]	4	9,174	934 (8.4)/943 (8.5)	0.99 (0.92–1.08)	0.844
Discontinue medication [16],[17],[32],[33]	4	9,874	179 (1.1)/161 (1.0)	0.96 (0.79–1.16)	0.663
**Total AEs associated with BP medication**					
Hypotension [16],[17],[29],[33]	4	14,138	76 (0.4)/16 (0.08)	4.16 (2.25–7.70)	<0.001
Dizziness [16],[17],[33]	3	6,229	220 (1.7)/193 (1.5)	1.15 (0.95–1.38)	0.148
Angioedema [16],[17]	2	5,844	7 (0.06)/5 (0.04)	1.40 (0.44–4.42)	0.565
Cough [17],[33]	2	1,496	14 (0.7)/11 (0.5)	0.67 (0.04–10.91)	0.775
Hyperkalemia [16],[33]	2	5,118	84 (0.7)/86 (0.7)	0.98 (0.73–1.32)	0.917
**Severe AEs associated with BP medication**					
Hypotension [16],[33]	2	5,118	17 (0.14)/3 (0.02)	2.19 (0.03–164.77)	0.723
Hyperkalemia [16],[33]	2	5,118	12 (0.1)/5 (0.04)	2.39 (0.20–28.59)	0.490
Renal failure [16],[33]	2	5,118	35 (0.3)/40 (0.3)	1.47 (0.26–8.23)	0.658
Angioedema [16]	1	4,733	6 (0.05)/4 (0.04)	1. 51 (0.43–5.33)	0.548
Syncope [16]	1	4,733	12 (0.1)/5 (0.04)	2.41 (0.85–6. 83)	0.088
Arrhythmia [16]	1	4,733	12 (0.1)/3 (0.03)	4.02 (1.13–14.21)	0.020

### Effects in Trial Subgroups

**Figure 5:**
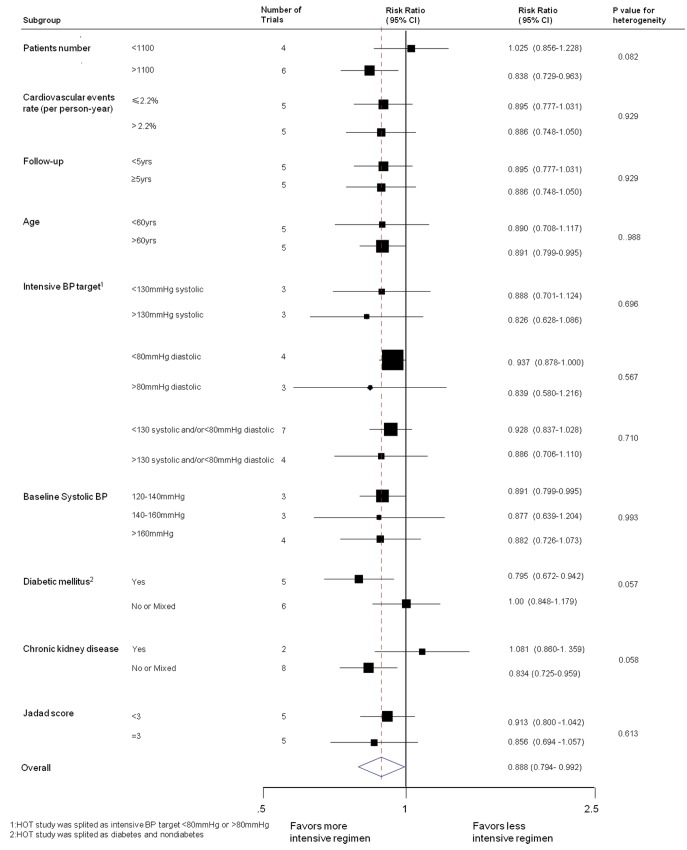
Effects of intensive BP lowering on the risk of major cardiovascular events in subgroups of trials.

There was no evidence that the observed effects of more intensive BP lowering regimens differed amongst trial subgroups defined according to a broad range of baseline characteristics (*p* for heterogeneity all *p*>0.05) (Figure 5). In particular, there was no clear evidence that the benefits of more intensive BP lowering varied by the starting mean baseline BP of the trial participants or the absolute level of the systolic or diastolic target set for the intensive group. Univariate meta-regression of intensive BP lowering on major cardiovascular outcomes according to the baseline characteristics also showed no evidence of heterogeneity (Table 4).

**Table 4:** Univariate meta-regression of intensive blood pressure lowering on major cardiovascular outcomes.

Variable	Studies	Scale	Proportional Change in RR (95% CI)	*p*-Value
Patients	10	Per 100	1.0003 (0.9981–1.0026)	0.739
Cardiovascular event rate (per person-year)	10	Per 1%	0.9224 (0.7955–1.0694)	0.243
Follow-up	10	Per year	1.0019 (0.9405–1.0672)	0.948
Age	10	Per 10 y	1.0058 (0.7959–1.2710)	0.956
Baseline systolic BP	10	Per 1 mmHg	0.9983 (0.9865–1.0103)	0.755

Formal statistical testing showed no obvious evidence of publication bias for the outcome of major vascular outcomes (*p*>0.05); however, the power to detect publication bias was limited as on only eight to ten studies were available for each comparison (Figure S1).

## Discussion

This meta-analysis, including more than 37,000 individuals amongst whom over 1,900 major vascular events were recorded, demonstrates a clear vascular benefit for more intensive BP lowering regimens aiming for lower BP targets. Major cardiovascular events were reduced by 11% and serious renal outcomes by 11% with specific benefit for a broad range of cardiovascular and renal outcomes, including myocardial infarction, stroke, albuminuria, and ESKD. However, there was no evidence to suggest that intensive BP treatment reduced or increased the risk of cardiovascular or noncardiovascular mortality. To the extent that it was possible to explore them, the observed beneficial effects did not appear to be attenuated by any characteristics of the patients involved or the BP regimens tested. Some adverse effects were more common in the intensively treated groups, but there was no suggestion that more intensive regimens were likely to result in net harm. In addition, the targets used in the most intensive BP control strategies were not associated with adverse cardiovascular outcomes or increased rates of death.

The findings from this overview are consistent with a recent analysis in patients with diabetes [37] but contrast with reports from some individual studies [16],[18] and a recent meta-analysis that have suggested no benefit from more intensive BP lowering regimens [15]. In both cases the most likely reason for this is the limited statistical power of the prior analyses. Few of the individual trials have recorded sufficient numbers of events and achieved large enough BP differences between randomized groups, to detect the most plausible effects of intensive BP control regimens on vascular outcomes. This is particularly so for the outcome of myocardial infarction, which is less strongly associated with BP than stroke, and therefore requires a much larger body of data to detect the anticipated effects. The prior much cited overview [15] had similar problems because the selective inclusion criteria, addressing a very narrow clinical question, meant that much applicable evidence was excluded. In this report, we approximately doubled the numbers of participants and events available for analysis, in large part because we were able to include new data from three large trials [16]–[18].

Key to interpreting the plausibility of the new findings presented here is an understanding of the broader clinical and epidemiological context. Associations observed in cohort studies and risk reductions seen in clinical trials of BP lowering versus control both provide indications of the magnitude of benefit that might be anticipated as a consequence of the 7.5/4.5-mmHg difference in BP seen in the current set of trials. The very close concordance between the expected benefits and those observed in this meta-analysis provides strong support for the validity of the current findings and argues for their wider generalisability.

It is now widely acknowledged that the observational association of BP with risk is direct and continuous to levels of BP far below the usual definition of hypertension [1]–[3]. Reported J-curve associations, seen mostly amongst patients with established disease, are likely to be attributable mostly to “reverse causation”—low BP is caused by the disease (e.g., prior heart attack) [38] and is associated with an increased risk of a poor outcome, but is not in itself the cause of the poor outcome. A number of recent post hoc analyses of clinical trial datasets have reignited concerns about the possibility of a J-curve for coronary disease at achieved systolic BP levels below 120 mmHg [39]–[41]. However, these analyses are nonrandomised in nature and need to be considered in light of the potential for confounding. The consistency of benefit at different baseline and achieved BP levels in this and other systematic reviews of all available evidence [4] suggests that confounding is indeed the reason for these observations.

We found evidence of benefit for clinically important microvascular outcomes with intensive BP lowering strategies. Specifically, the risk of ESKD was reduced by 11%. Similarly, trials in people with diabetes showed evidence of a reduced incidence of microalbuminuria and a trend towards a reduced incidence of retinopathy. Taken together, these results provide substantial reassurance about the renal safety of intensive BP lowering and suggest benefit for microvascular outcomes is likely.

The present overview did not provide especially clear evidence about the effects of more intensive BP control on side effects because the quantity of available data was limited. Adverse events and serious adverse events were not increased overall, but an increased frequency of hypotension was observed. Of note, absolute rates of serious side effects appeared to be low and infrequently led to discontinuation of the intensive BP lowering strategy, although reporting of these events was suboptimal so some caution must be exercised in interpreting these results. These findings would suggest that lower targets for BP are likely to be achievable for many individuals and that there would be significant net benefit to population health if the strategy were widely implemented, although more precise data regarding the totality of adverse outcomes would be important in clarifying the remaining uncertainty in this regard.

This overview benefits from the rigorous methodology used, the homogeneity of the individual trial results summarized by the meta-analyses, and the consistent effects observed across a range of macro- and microvascular disease outcomes. All serve to provide reassurance about the likely validity of the primary conclusions. Chief among the limitations are the moderate number and size of trials available, the heterogeneity of participants in included trials, and in particular the few data to describe directly the effects of intensive BP lowering amongst individuals with uncomplicated hypertension. Most trials included in this study included participants with additional cardiovascular risk factors, including diabetes or CKD, which also limits the generalisability of the findings. Additionally, the subgroup analyses are based on the study characteristics rather than individual patient data (IPD). An IPD meta-analysis would provide important additional information. Finally, although this analysis suggests that BP targets at 130/80 or lower are likely to produce additional overall benefit, there is insufficient data to confirm a specific BP threshold. These analyses gain from the inclusion of analyses of renal outcomes. With ageing of the population, CKD is becoming an increasingly large cause of disease burden and documenting the effects on hard renal outcomes is central to estimating the overall balance of risks and benefits.

A range of research questions arise from this work, perhaps most importantly how best to achieve and maintain greater BP reductions in high-risk patients, particularly given the relatively modest BP differences between the randomized groups achieved on average in the completed trials. Large and rapid reductions may be less well tolerated, particularly if hypertension has been severe and longstanding, but the optimal ways to achieve this while maintaining adherence are still uncertain. It is apparent that low-dose combinations will be an important part of this solution [42]–[44] but other approaches to improve treatment rates and adherence will be required.

In conclusion, these overviews provide support for clinical guidelines advocating more intensive BP lowering amongst high-risk patient groups, although the limitations of the available data mean that the results should be generalized with some caution. Whilst few large-scale randomized trials have been done to evaluate the effects of intensive BP lowering amongst patients with uncomplicated hypertension, and more data would clearly be helpful in defining the groups most likely to benefit as well as to suffer adverse effects, the totality of the current evidence suggests that benefits are likely to be greater than harms. BP lowering to below current thresholds may achieve additional benefits and reduce the burden of cardiovascular morbidity and mortality for many patients. If our data are applied to hypertensive patients at high cardiovascular risk with an annual cardiovascular event rate of about 2%, the available data suggest that among every thousand such people, intensive BP lowering could prevent two of the 20 cardiovascular events expected to occur each year, while increasing one severe hypotension event.

## Supporting Information

Figure S1 Begg's funnel plot for the assessment of publication bias in studies examining the effects of intensive BP lowering on major cardiovascular outcomes (Egger's test *p* = 0.668), stroke (*p* = 0.125), myocardial infarction (*p* = 0.166), and end stage of kidney disease (*p* = 0.555).
(TIF)

Table S1 Quality analyses of the trials included in the systematic review and meta-analysis.
(DOCX)

Text S1 PRISMA checklist.
(DOCX)

Text S2 Search strategy.
(DOCX)

Text S3 Study protocol.
(DOC)

## Abbreviations

BP: blood pressure
CHD: coronary heart disease
CKD: chronic kidney disease
ESKD: end stage kidney disease
RR: relative risk
