# Supplementary figures and images for: Effects of Intensive Blood Pressure Lowering on Cardiovascular and Renal Outcomes: A Systematic Review and Meta-Analysis
Source: PLoS Med. 2012 Aug 21;9(8):e1001293. doi: 10.1371/journal.pmed.1001293 (PMC3424246; doi:10.1371/journal.pmed.1001293)

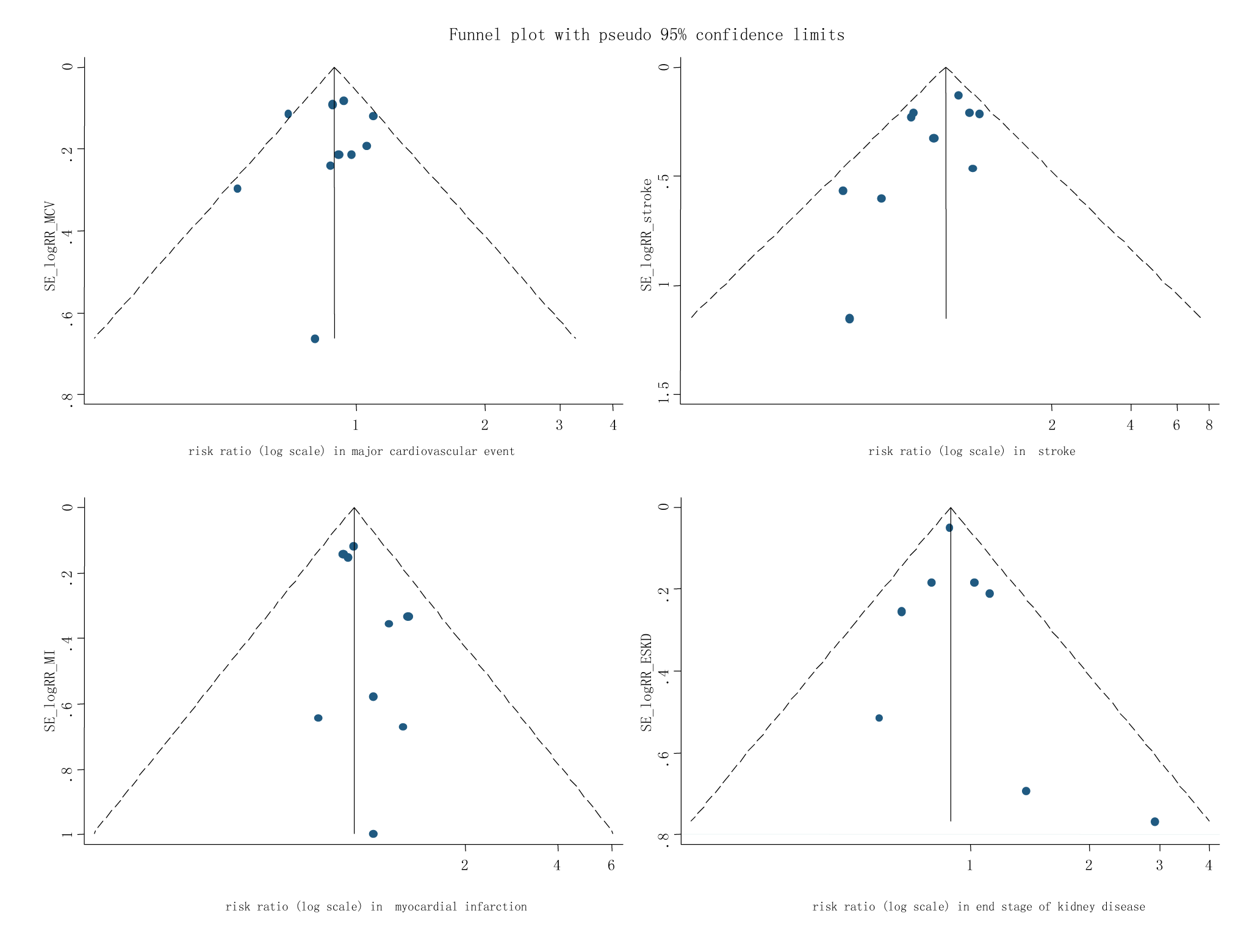

Supplement: Figure S1 — Begg's funnel plot for the assessment of publication bias in studies examining the effects of intensive BP lowering on major cardiovascular outcomes (Egger's test p = 0.668), stroke (p = 0.125), myocardial infarction (p = 0.166), and end stage of kidney disease (p = 0.555). (TIF) [file pmed.1001293.s001.tif]
