# Supplementary material for: Effects of Intensive Blood Pressure Lowering on Cardiovascular and Renal Outcomes: A Systematic Review and Meta-Analysis
Source: PLoS Med. 2012 Aug 21;9(8):e1001293. doi: 10.1371/journal.pmed.1001293 (PMC3424246; doi:10.1371/journal.pmed.1001293)
Supplement: Table S1 — Quality analyses of the trials included in the systematic review and meta-analysis. (DOCX) [file pmed.1001293.s002.docx]

| **Table S1: Quality analyses of the trials included in the systematic review and meta-analysis** | | | | | | | |
| --- | --- | --- | --- | --- | --- | --- | --- |
| Study/author  (year) | Adequate sequence generation | Allocation concealment | **blinding** | | | Incomplete outcome data addressed | Free of selective outcome reporting |
|  |  |  | outcome assessors | personnel | participants |  |  |
| Toto (1995) | unclear | unclear | unclear | no | no | unclear | unclear |
| HOT (1998) | yes | yes | yes | no | no | unclear | yes |
| UKPDS-HDS (1998) | yes | yes | yes | no | no | unclear | yes |
| ABCD (H) (2000) | yes | yes | yes | no | yes | unclear | yes |
| ABCD (N) (2001) | yes | yes | yes | no | yes | unclear | yes |
| AASK (2010) | yes | yes | yes | no | no | yes | yes |
| MDRD (2005) | yes | yes | unclear | no | no | yes | yes |
| REIN-2 (2005) | yes | yes | ns | no | no | yes | unclear |
| ABCD (2V) (2006) | yes | yes | yes | no | yes | yes | unclear |
| JATOS (2008) | yes | yes | yes | no | no | yes | unclear |
| Cardio-Sis (2009) | yes | yes | yes | no | no | yes | yes |
| ESCAPE (2009) | yes | ns | unclear | no | no | yes | unclear |
| ACCORD (2010) | unclear | yes | unclear | no | no | unclear | yes |
| VANLISH (2010) | yes | unclear | yes | no | no | yes | yes |
| * The ABCD 2V study was terminated after 5 years due to the funding constrains. | | | | | | | |
