# Supplementary material for: Effects of Intensive Blood Pressure Lowering on Cardiovascular and Renal Outcomes: A Systematic Review and Meta-Analysis
Source: PLoS Med. 2012 Aug 21;9(8):e1001293. doi: 10.1371/journal.pmed.1001293 (PMC3424246; doi:10.1371/journal.pmed.1001293)
Supplement: Text S2 — Search strategy. (DOCX) [file pmed.1001293.s004.docx]

**Text S2: searching strategy**

**MEDLINE (OVID) AND**

1. exp antihypertensive agents/

2. (antihypertensive$ adj (agent$ or drug)).tw.

3. chlorothiazide.tw.

4. chlorthalidone.tw.

5. hydralazine.tw.

6. hydrochlorothiazide.tw.

7. indapamide.tw.

8. minoxidil.tw.

9. exp angiotensin converting enzyme inhibitors/

10. captopril.tw.

11. enalapril.tw.

12. cilazapril.tw.

13. enalaprilat.tw.

14. fosinopril.tw.

15. lisinopril.tw.

16. perindopril.tw.

17. ramipril.tw.

18. saralasin.tw.

19. teprotide.tw.

20. exp losartan/

21. losartan.tw.

22. imidazole$.tw.

23. irbesartan.tw.

24. candesartan.tw.

25. eprosartan.tw.

26. valsartan.tw.

27. olmesartan.tw.

28. telmisartan.tw.

29. (ace adj2 inhibitor$).tw.

30. (angiotensin adj2 receptor antagonist$).tw.

31. exp calcium channel blockers/

32. amlodipine.tw.

33. diltiazem.tw.

34. felodipine.tw.

35. nicardipine.tw.

36. nifedipine.tw.

37. nimodipine.tw.

38. nisoldipine.tw.

39. nitrendipine.tw.

40. verapamil.tw.

41. exp adrenergic beta-antagonists/

42. alprenolol.tw.

43. atenolol.tw.

43a.carvedilol.tw

43b. bisoprolol.tw

44. metoprolol.tw.

45. nadolol.tw.

46. oxprenolol.tw.

47. pindolol.tw.

48. propranolol.tw.

49. exp adrenergic alpha-antagonists/

50. labetalol.tw.

51. prazosin.tw.

52. beta block$.tw.

53. exp diuretics/

54. spironolactone.tw.

55. triamterene.tw.

56. bumetanide.tw.

57. furosemide.tw.

58. or/1-57

59.exp Clinical Trial/

60. exp Random Allocation/

61. exp Single Blind Method/

62. exp Double Blind Method/

63. (random$ adj5 trial$).tw.

64. (random$ adj5 allocation$).tw.

65. (Blind$ adj5 method$).tw.

66. or/59-65

67. (target level).mp

68. (blood pressure adj6 target).mp

69. (BP adj6 target).mp

70. (blood pressure adj6 goal).mp

71. (BP adj6 goal).mp

72. (intensi$ adj6 treatment).mp

73.( intensi$ adj6 control).mp

74.( intensi$ adj6 lowering).mp

75. (intensi$ adj6 blood pressure).mp

76. (intensi$ antihypertensive).mp

77. (tight adj6 control).mp

78. (tight adj6 blood pressure).mp

79. (strict adj6 control).mp

80. (strict adj6 blood pressure).mp

81. or/67-80

82. 58 and 66 and 81

**COCHRANE CONTROLLED TRIALS**

1. antihypertensive agents explode all trees

2. (antihypertensive$ adj (agent$ or drug))

3. chlorothiazide

4. chlorthalidone

5. hydralazine

6. hydrochlorothiazide

7. indapamide

8. minoxidil

9. angiotensin converting enzyme inhibitors explode all trees

10. captopril

11. enalapril

12. cilazapril

13. enalaprilat

14. fosinopril

15. lisinopril

16. perindopril

17. ramipril

18. saralasin

19. teprotide.

20. losartan explode all trees

21. losartan

22. imidazole

23. irbesartan

24. candesartan

25. eprosartan

26. valsartan

27. olmesartan

28. telmisartan

29. (ace adj2 inhibitor$)

30. (angiotensin adj2 receptor antagonist$)

31. calcium channel blockers explode all trees

32. amlodipine

33. diltiazem

34. felodipine

35. nicardipine

36. nifedipine

37. nimodipine

38. nisoldipine

39. nitrendipine

40. verapamil

41. adrenergic beta-antagonists explode all trees

42. alprenolol

43. atenolol

43a. Carvedilol

43b. bisoprolol

44. metoprolol

45. nadolol

46. oxprenolol

47. pindolol

48. propranolol

49. adrenergic alpha-antagonists explode all trees

50. labetalol

51. prazosin

52. beta block

53. diuretics explode all trees

54. spironolactone

55. triamterene

56. bumetanide

57. furosemide

58. or/1-57

59. (target level).mp

60. (blood pressure adj6 target).mp

61. (BP adj6 target).mp

62. (blood pressure adj6 goal).mp

63. (BP adj6 goal).mp

64. (intensi$ adj6 treatment).mp

65.( intensi$ adj6 control).mp

66.( intensi$ adj6 lowering).mp

67. (intensi$ adj6 blood pressure).mp

68. (intensi$ antihypertensive).mp

69. (tight adj6 control).mp

70. (tight adj6 blood pressure).mp

71. (strict adj6 control).mp

72. (strict adj6 blood pressure).mp

73. or/59-72

74. 58 and 73

**EMBASE**

1. antihypertensive agents

2. chlorothiazide

3. chlorthalidone

4. hydralazine

5. hydrochlorothiazide

6. indapamide

7. minoxidil

8. losartan

8. imidazole

10. irbesartan

11. candesartan

12. eprosartan

13. valsartan

14. olmesartan

15. telmisartan

16. angiotensin converting enzyme inhibitors

17. captopril

18. enalapril

19. fosinopril

20. lisinopril

21. perindopril

22. ramipril

23. saralasin

24. teprotide

25. Angiotensin 2 Receptor Antagonist

26. Angiotensin Receptor Antagonist

27. Angiotensin II Antagonist

28. AT 2 receptor blocker

29. AT 2 receptor antagonist

30. angiotensin receptor antagonist

31. Calcium Channel Blockers

32. amlodipine

33. diltiazem

34. felodipine

35. nicardipine

36. nifedipine

37. nimodipine

38. nisoldipine

39. nitrendipine

40. verapamil

41. adrenergic beta-antagonists

42. alprenolol

43. atenolol

44 carvedilol

45. bisoprolol

46. metoprolol

47. nadolol

48. oxprenolol

49. pindolol

50. propranolol

51. adrenergic alpha-antagonists/

52. labetalol

53. prazosin

54. diuretics

55. spironolactone

56. triamterene

57. bumetanide

58. furosemide

59. clinical and trial

60. randomized and controlled and trial

61. random and allocation

62. single blind and method

63. double blind and method

64. target level

65. target blood pressure

66. Target systolic blood pressure

67. Target diastolic blood pressure

68. Intensive treatment

69. Intensive blood pressure treatment

70. Intensive antihypertensive treatment

71. Intensive control

72. Intensive blood pressure control

73. Tight control

74. Tight blood pressure control

75. Strict control

76. Strict blood pressure control

77. or/1-58

78. or/59-63

79. or/64-76

80. #77 and #78 and #79
