# Supplementary material for: Effects of Intensive Blood Pressure Lowering on Cardiovascular and Renal Outcomes: A Systematic Review and Meta-Analysis
Source: PLoS Med. 2012 Aug 21;9(8):e1001293. doi: 10.1371/journal.pmed.1001293 (PMC3424246; doi:10.1371/journal.pmed.1001293)
Supplement: Text S3 — Study protocol. (DOC) [file pmed.1001293.s005.doc]

PROTOCOL First drafted in April 2010

Updated in July 2011

**Effect of lower blood pressure target for the macro- or micro-vascular event: systematic review and meta-analysis**

Jicheng Lv, Vlado Perkovic

The George institute for international health

**Objectives and aims:**

To determine if a lower blood pressure (BP) target is associated with reduction of mortality and morbidity in patients with hypertension.

**Background**

Cohort studies have compelling shown that risk for cardiovascular events in a log-linear fashion as BP rises above 115mmHg.^1,2^ Such data suggest that lower BP target even below the standard target 140/90mmHg that most guidelines recommended might benefit patients with hypertension. However, observational data are subject to confounding and optimum treatment threshold should be based on RCTs. Recently several large convincing RCTs support the lower treatment goal in patients with high risk for macrovascular (eg with cardiovascular disease) or microvascular (eg diabetic nephropathy).^3-6^

A recent Cochrane systematic review involve more than 22,000 patients with a 7-mmHg difference in SBP between lower (<135/85) and usual target (<140-160/90mmHg), however, did not show significant differences in CV outcomes including stroke, MI and mortality. However we must consider this meta-analysis cautiously, at least for several reasons

First, several large trials are not involve in this meta-analysis including BBB(1994) Cardio-Sis (2009) and ACCORD (2010). Cardio-Sis study involving 1111 nondiabetic patients patients revealed that a tight BP control target below 130mmHg reduce the composite of cardiovascular event rate by 50% (HR 0.50). The recent ACCORD trial in which a lower blood pressure target of systemic BP <120mmHg only showed a trend for reduced major CV events yet was not significant. However the BP target reduced stroke (HR 0.59) and new onset of diabetic nephropathy.

Second most trials in this meta-analysis are small trials. More than 90% patients and 75% endpoint event were from HOT study. In fact the HOT study support a more intensive BP control in diabetic nephropathy. This result also differed from an earlier meta-analysis by the BP Lowering Treatment Titlists Collaboration (BPLTTC) which demonstrated intensive BP control reduced the major cardiovascular events. The reason for this difference that the Cochrane meta-analysis did not include the UKPDS 38 study. The Cochrane review excluded it because the BP target in UKPDS study was 150/85, not an intensive target . In fact the mean achieved BP in HOT study(tight vs less tight: 140.6 vs 143.7mmHg) and UKPDS study (144 vs 154mmHg) were similar.

Third, the achieved mean BP reduction between the intervention and control group are different (from -3.15 to -13 mmHg) in these trials are different. The BPLTTC review had clearly demonstrated that the Size of blood pressure reduction was an important determinant of outcome and thus the Cochrane just pool them also maybe misleading.

Clearly we need a systematic review with including these new trials and comprehensive analysis to get a critical answer for the BP target question.

**Research Plan:**

**A) Methods of the review**

The study will be conducted according to the PRISMA statement for the conduct of meta-analyses of intervention studies.

**B) Data sources:**

Relevant randomized controlled trials will be identified by computerized searches from the following data sources without language restriction: MEDLINE via Ovid (from 1950 through July 2011), PubMed, EMBASE (from 1966 through July 2011), and Cochrane Library databases. Reference lists in relevant trials, review articles (3-8) and guidelines for hypertension (JNC 7, WHO/ISH 2003, K/DOQI 2004, BHS 2004, ESH-ESC 2007, AHA 2007) will be searched as well.

**C) Study selection:**

**Types of studies:**

Randomized controlled trials, examining benefits and harms of intensive BP control to a lower target.

**Types of participants:**

Inclusion criteria: Patients (not including gestational hypertension) with elevated BP documented in a standard way or patients already received BP lowering agents, or those with cardiovascular risk (e.g. diabetes or chronic kidney disease) that need receive intensive BP lowering

Exclusion criteria: No exclusion criteria:

**Type of intervention:**

For different blood pressure targets.

**Type of outcome measures*:***

1. All cause Mortality
2. Cardiovascular mortality
3. Major coronary event including fatal/non fatal MI, unstable angina
4. Stroke, fatal or non fatal
5. Heart failure, fatal or non fatal
6. Composite of cardiovascular event including non fatal myocardial infarction, non fatal stroke, cardiovascular death
7. Renal failure including ESKD, doubling scrum creatinine
8. Other renal outcomes including new onset of microalbuminuria or worsening of albuminuria in diabetes
9. Mean blood pressure lowering
10. Any adverse events, including adverse event related to hypotension

**D) The search strategy:**

MEDLINE (OVID) AND

1. exp antihypertensive agents/

2. (antihypertensive$ adj (agent$ or drug)).tw.

3. chlorothiazide.tw.

4. chlorthalidone.tw.

5. hydralazine.tw.

6. hydrochlorothiazide.tw.

7. indapamide.tw.

8. minoxidil.tw.

9. exp angiotensin converting enzyme inhibitors/

10. captopril.tw.

11. enalapril.tw.

12. cilazapril.tw.

13. enalaprilat.tw.

14. fosinopril.tw.

15. lisinopril.tw.

16. perindopril.tw.

17. ramipril.tw.

18. saralasin.tw.

19. teprotide.tw.

20. exp losartan/

21. losartan.tw.

22. imidazole$.tw.

23. irbesartan.tw.

24. candesartan.tw.

25. eprosartan.tw.

26. valsartan.tw.

27. olmesartan.tw.

28. telmisartan.tw.

29. (ace adj2 inhibitor$).tw.

30. (angiotensin adj2 receptor antagonist$).tw.

31. exp calcium channel blockers/

32. amlodipine.tw.

33. diltiazem.tw.

34. felodipine.tw.

35. nicardipine.tw.

36. nifedipine.tw.

37. nimodipine.tw.

38. nisoldipine.tw.

39. nitrendipine.tw.

40. verapamil.tw.

41. exp adrenergic beta-antagonists/

42. alprenolol.tw.

43. atenolol.tw.

43a. carvedilol.tw

43b. bisoprolol.tw

44. metoprolol.tw.

45. nadolol.tw.

46. oxprenolol.tw.

47. pindolol.tw.

48. propranolol.tw.

49. exp adrenergic alpha-antagonists/

50. labetalol.tw.

51. prazosin.tw.

52. beta block$.tw.

53. exp diuretics/

54. spironolactone.tw.

55. triamterene.tw.

56. bumetanide.tw.

57. furosemide.tw.

58. or/1-57

1. exp Clinical Trial/
2. exp Random Allocation/
3. exp Single Blind Method/
4. exp Double Blind Method/
5. (random$ adj5 trial$).tw.
6. (random$ adj5 allocation$).tw.
7. (Blind$ adj5 method$).tw.
8. or/59-65

67. (target level).mp

68. (blood pressure adj6 target).mp

69. (BP adj6 target).mp

70. (blood pressure adj6 goal).mp

71. (BP adj6 goal).mp

72. (intensi$ adj6 treatment).mp

73.( intensi$ adj6 control).mp

74.( intensi$ adj6 lowering).mp

75. (intensi$ adj6 blood pressure).mp

76. (intensi$ antihypertensive).mp

77. (tight adj6 control).mp

78. (tight adj6 blood pressure).mp

79. (strict adj6 control).mp

80. (strict adj6 blood pressure).mp

81. or/67-80

82. 58 and 66 and 81

COCHRANE CONTROLLED TRIALS

1. antihypertensive agents explode all trees

2. (antihypertensive$ adj (agent$ or drug))

3. chlorothiazide

4. chlorthalidone

5. hydralazine

6. hydrochlorothiazide

7. indapamide

8. minoxidil

9. angiotensin converting enzyme inhibitors explode all trees

10. captopril

11. enalapril

12. cilazapril

13. enalaprilat

14. fosinopril

15. lisinopril

16. perindopril

17. ramipril

18. saralasin

19. teprotide.

20. losartan explode all trees

21. losartan

22. imidazole

23. irbesartan

24. candesartan

25. eprosartan

26. valsartan

27. olmesartan

28. telmisartan

29. (ace adj2 inhibitor$)

30. (angiotensin adj2 receptor antagonist$)

31. calcium channel blockers explode all trees

32. amlodipine

33. diltiazem

34. felodipine

35. nicardipine

36. nifedipine

37. nimodipine

38. nisoldipine

39. nitrendipine

40. verapamil

41. adrenergic beta-antagonists explode all trees

42. alprenolol

43. atenolol

43a. Carvedilol

43b. bisoprolol

44. metoprolol

45. nadolol

46. oxprenolol

47. pindolol

48. propranolol

49. adrenergic alpha-antagonists explode all trees

50. labetalol

51. prazosin

52. beta block

53. diuretics explode all trees

54. spironolactone

55. triamterene

56. bumetanide

57. furosemide

58. or/1-57

59. (target level).mp

60. (blood pressure adj6 target).mp

61. (BP adj6 target).mp

62. (blood pressure adj6 goal).mp

63. (BP adj6 goal).mp

64. (intensi$ adj6 treatment).mp

65.( intensi$ adj6 control).mp

66.( intensi$ adj6 lowering).mp

67. (intensi$ adj6 blood pressure).mp

68. (intensi$ antihypertensive).mp

69. (tight adj6 control).mp

70. (tight adj6 blood pressure).mp

71. (strict adj6 control).mp

72. (strict adj6 blood pressure).mp

73. or/59-72

74. 58 and 73

EMBASE

1. antihypertensive agents

2. chlorothiazide

3. chlorthalidone

4. hydralazine

5. hydrochlorothiazide

6. indapamide

7. minoxidil

8. losartan

8. imidazole

10. irbesartan

11. candesartan

12. eprosartan

13. valsartan

14. olmesartan

15. telmisartan

16. angiotensin converting enzyme inhibitors

17. captopril

18. enalapril

19. fosinopril

20. lisinopril

21. perindopril

22. ramipril

23. saralasin

24. teprotide

25. Angiotensin 2 Receptor Antagonist

26. Angiotensin Receptor Antagonist

27. Angiotensin II Antagonist

28. AT 2 receptor blocker

29. AT 2 receptor antagonist

30. angiotensin receptor antagonist

31. Calcium Channel Blockers

32. amlodipine

33. diltiazem

34. felodipine

35. nicardipine

36. nifedipine

37. nimodipine

38. nisoldipine

39. nitrendipine

40. verapamil

41. adrenergic beta-antagonists

42. alprenolol

43. atenolol

44 carvedilol

45. bisoprolol

46. metoprolol

47. nadolol

48. oxprenolol

49. pindolol

50. propranolol

51. adrenergic alpha-antagonists/

52. labetalol

53. prazosin

54. diuretics

55. spironolactone

56. triamterene

57. bumetanide

58. furosemide

59. clinical and trial

60. randomized and controlled and trial

61. random and allocation

62. single blind and method

63. double blind and method

64. target level

65. target blood pressure

66. Target systolic blood pressure

67. Target diastolic blood pressure

68. Intensive treatment

69. Intensive blood pressure treatment

70. Intensive antihypertensive treatment

71. Intensive control

72. Intensive blood pressure control

73. Tight control

74. Tight blood pressure control

75. Strict control

76. Strict blood pressure control

77. or/1-58

78. or/59-63

79. or/64-76

76. #77 and #78 and #79

**Reference**

1. MacMahon S, Peto R, Cutler J, Collins R, Sorlie P, Neaton J, Abbott R, Godwin J, Dyer A, Stamler J. Blood pressure, stroke, and coronary heart disease. Part 1, Prolonged differences in blood pressure: prospective observational studies corrected for the regression dilution bias. Lancet. 1990 Mar 31;335(8692):765-74.
2. Lewington S, Clarke R, Qizilbash N, Peto R. Collins R. Age-specific relevance of usual blood pressure to vascular mortality: a meta-analysis of individual data for one million adults in 61 prospective studies. Prospective studies Collaboration. Lancet 2002;360:1903-1913
3. Eastacio RO, Jeﬀ ers BW, Hiatt WR, Biggerstaﬀ SL, Giﬀ ord N, Schrier RW. The effects of nisoldipine as compared with enalapril on cardiovascular outcomes in patients with non-insulin-dependent diabetes and hypertension. N Engl J Med 1998; 338: 645–52.
4. The Heart Outcomes Prevention Evaluation Study Investigators. Effects of an angiotensin-converting enzyme inhibitor, ramipril, on cardiovascular events in high risk patients. N Engl J Med 2000; 342: 145–53.
5. ADVANCE Collaborative Group. Effects of a ﬁxed combination of perindopril and indapamide on macrovascular and microvascular outcomes in patients with type 2 diabetes melitus (the ADVANCE trial): a randomised controled trial. Lancet 2007; 370: 829–40
6. Verdecchia P, Staessen JA, Angeli F, de Simone G, Achilli A, Ganau A, Mureddu G, Pede S, Maggioni AP, Lucci D, Reboldi G; Cardio-Sis investigators. Usual versus tight control of systolic blood pressure in non-diabetic patients with hypertension (Cardio-Sis): an open-label randomised trial. Lancet 2009;374:525-33
7. Arguedas JA, Perez MI, Wright JM. Treatment blood pressure targets for hypertension. Cochrane Database Syst Rev 2009; 3: CD004349.pub2.
8. Turnbull F; Blood Pressure Lowering Treatment Trialists’ Collaboration. Effects of different blood-pressure-lowering regimens on major cardiovascular events: results of prospectively-designed overviews of randomised trials. Lancet. 2003;362:1527-35.
9. UK Prospective Diabetes Study Group. Tight blood pressure control and risk of macrovascular and microvascular complications in type 2 diabetes: UKPDS 38. UK Prospective Diabetes Study Group. BMJ. 1998;317:703-13.
10. Moher D, Cook DJ, Eastwood S, Olkin I, Rennie D, Stroup DF. Improving the quality of reports of meta-analyses of randomised controlled trials: the QUOROM statement. Quality of Reporting of Meta-analyses. Lancet. 1999; 354:1896-900.
